# Estimating the Health-Related Quality of Life of Twitter Users Using Semantic Processing

**DOI:** 10.3233/SHTI190388

**Published:** 2019-08-21

**Authors:** Karthik V. Sarma, Brennan M. R. Spiegel, Mark W. Reid, Shawn Chen, Raina M. Merchant, Emily Seltzer, Corey W. Arnold

**Affiliations:** a Department of Radiological Sciences, University of California, Los Angeles, Los Angeles, CA, USA; b Center for Outcomes Research and Education, Cedars-Sinai Medical Center, Los Angeles, CA, USA; c Penn Medicine Center for Digital Health, University of Pennsylvania, Philadelphia, PA, USA

**Keywords:** Quality of Life, Social Media, Natural Language Processing

## Abstract

Social media presents a rich opportunity to gather health information with limited intervention through the analysis of completely unstructured and unlabeled microposts. We sought to estimate the health-related quality of life (HRQOL) of Twitter users using automated semantic processing methods. We collected tweets from 878 Twitter users recruited through online solicitation and in-person contact with patients. All participants completed the four-item Centers for Disease Control Healthy Days Questionnaire at the time of enrollment and 30 days later to measure “ground truth” HRQOL. We used a combination of document frequency analysis, sentiment analysis, topic analysis, and concept mapping to extract features from tweets, which we then used to estimate dichotomized HRQOL (“high” vs. “low”) using logistic regression. Binary HRQOL status was estimated with moderate performance (AUC=0.64). This result indicates that free-range social media data only offers a window into HRQOL, but does not afford direct access to current health status.

## Introduction

Social media platforms are increasingly used for clinical research. Investigators now harness social media data for many purposes, including recruiting patients into clinical trials [1], measuring mood and sentiments [2], educating patients about social and medical concerns [3], and many others [4–9]. Although these applications expand the ability to perform health outcomes research on a broad scale, there is currently no reliable technique to measure patient reported outcomes (PROs), such as health related quality of life (HRQOL), using social media data.

The availability of a psychometrically valid, automatically generated social media PRO instrument would offer researchers and clinicians a new method to measure the “real life” effectiveness of interventions, behaviors, and therapies outside the confines of traditional research. Ideally, such a PRO would not require explicitly prompting users to speak specifically about health-related issues. Instead, the PRO would rely on existing social media posts and then estimate HRQOL using techniques such as natural language processing (NLP) and sentiment analysis.

An important step toward using social media for HRQOL estimation is to develop a method for translating diverse, unprompted, unstructured social media posts into information useable by clinicians and researchers. Given concerns about the representativeness and validity of social media data, there are limitations to measuring “true” HRQOL using social media analytics. Specifically, social media users may be systematically different from non-users, and even among users, many likely display a version of themselves that does not reflect their true functional state. Nevertheless, it would be useful to develop a method of extracting meaningful information from social media posts that operates within these limitations.

Several previous studies have looked at whether information about various components of an individual’s health status could be inferred from unprompted social media use. For example, one study found that language used in Facebook posts could predict depression [10]. Another study found that aggregated Twitter data could predict county-level mortality from heart disease [11]. Others still have looked at PTSD [12] and county-level life satisfaction [13] with positive results. To the best of our knowledge, however, this is the first study that has tried to directly predict HRQOL measured by a validated PRO instrument using unprompted social media data.

In the present study, we collected social media posts from users of Twitter (http://www.twitter.com/), a web- and smartphone-based tool for users to express comments in short statements (“Tweets”), often appended with images or embedded with links to websites. We contacted users and administered a brief, empirically validated questionnaire that assesses HRQOL. We then used multiple analytic methods to develop predictors of their ground-truth HRQOL scores using data solely from their “Tweets”. We hypothesized that, despite the limitations of free-range social media data, HRQOL could be classified with a degree of accuracy greater than chance.

## Methods

### Study Overview

We recruited a cohort of 1,831 Twitter users between May and December of 2015 through a combination of online solicitation and in-person contact. All participants were asked to complete the four-item Centers for Disease Control Healthy Days Questionnaire (CDC-4) questionnaire [14] at the time of enrollment and 30 days later to measure “ground truth” HRQOL. We then collected their tweets over a period of 60 days: 30 days before and after enrollment. Next, we collected and analyzed the tweets to determine characteristics that distinguish users with low or high HRQOL, using the CDC-4 as a gold standard. Of the 1,831 participants recruited, 878 had at least one accessible tweet during the analysis period, and the remaining participants were not used for analysis.

### Participant Recruitment

We recruited participants through three mechanisms. First, we recruited general Twitter users by tweeting announcements through our institutional Twitter account (@CedarsSinai). Second, we recruited a general population sample through Cint, a survey research firm that recruited a sample of Twitter users from its survey cohort. Third, we supplemented our cohort of general Twitter users with a defined group of patients seeking care by recruiting individuals seen at the University of Pennsylvania Emergency Department for non-life-threatening, ambulatory conditions. Subjects in all cohorts were required to be 18 years of age or older and able to read English. Participants were entered into a drawing for one of two randomly selected $500 USD prizes. Participants with Twitter accounts who consented to participate completed the CDC-4 and provided their Twitter handle. Additional information about the University of Pennsylvania cohort has been published previously [15].

### Study Measure

The CDC-4 Healthy Days Questionnaire [14] is a brief measure that assesses a number of factors, including general HRQOL on a five-point scale. This measure has been rigorously validated, demonstrating criterion validity with the Short Form 36-item health survey (SF-36) [16] as well as content, construct, and predictive validity, internal consistency, and test-retest reliability across diverse populations. The measure was chosen for its simplicity, reducing respondent burden to a minimum.

### Acquisition and Preprocessing of Twitter Data

All tweets were acquired from the Twitter website. Tweets collected from each participant were segregated into two “collection waves”: tweets from the 30 days prior to enrollment, and tweets from the 30 days including and subsequent to enrollment.

In order to reduce the proportion of marketing-related tweets in our dataset, we filtered out tweets containing any of a set of key phrases (any URLs, or the strings “sponsored”, “my echo”, “#mpoints”, “giveaway”, “@youtube”, “giftcard”, and “gift card.”) that were determined to be associated in our dataset with marketing tweets by manual review of a random tweet sample. In addition, any users who tweeted more than 300 times in a given 30-day collection wave were removed from analysis in that period. This threshold was determined manually in order to remove accounts that were predominated by spam.

We then applied an initial preprocessing pipeline consisting of custom filters as well as Natural Language Tool Kit (NLTK) [17] tools to all tweets. The pipeline consisted of the following steps:

1. Unicode normalization form C (NFC) was applied
2. The Penn Treebank tokenizer was applied to generate tokens
3. Mention and reply tokens (e.g., @username) were removed
4. All tokens were converted to lower case
5. Repeated characters were removed iteratively until either no additional repeat characters existed or the token was transformed into a word in WordNet [18]
6. A Lancaster [19] stemmer was applied to each token

After preprocessing, we created “tweet sets” consisting of the concatenation of all of the tweets from each of the two collection waves (thus creating two tweet sets per participant). We then applied a term frequency-inverse document frequency (TF-IDF) transformer to each tweet set. For each of these tweet sets, we also computed several additional features. We fed each acquired tweet in each document set into the SentiStrength sentiment analysis tool, a validated opinion mining toolkit that computes positive and negative sentiment scores [20–22]. Then, we computed the mean, median, standard deviation, maximum, and minimum of the SentiStrength scores across all tweets in the set, and added them to the set’s data vector. We also added the number of tweets in the set to the vector.

In order to conduct semantic processing on the large volume of tweets, we utilized Latent Dirichlet Allocation (LDA), an automated method for discovering semantic themes in unstructured text [23]. For each concatenated tweet set, a vector representing the topics contained in the set was generated and added to the set’s data vector. Selected examples for demonstration are presented in Table 1.

Finally, we used the cTAKES processing engine [24] to map words in each tweet to concepts found in the Unified Medical Language System (UMLS) Metathesaurus and to detect concept negation. Instances in which the concepts were negated were considered to be separate concepts from non-negated versions. After determining the list of all concepts present in our dataset, for each 30-day tweet set, we created a data vector representing whether or not each concept was present or absent in the set. We then added this vector to the set’s data vector.

### Statistical Analysis

We first dichotomized survey responses to HRQOL question 1a – a single 5-level global measure of HRQOL – into a “high” (>3) or “low” (<3) HRQOL binary variable; responses of exactly 3 were discarded. Next, we applied several machine learning techniques to produce a model that predicted this variable using the input vectors described above and 5-fold cross-validation. We employed the following techniques: standard logistic regression, random forest classifiers, and bagging generalized linear models (GLMs) trained using stochastic gradient descent (SGD).

We also applied a range of explicit and implicit feature selection techniques. Explicit methods included the use of a variance threshold for each column, principle component analysis (PCA), selecting the K best columns based on a univariate t-test, a unit origin transformer, and class balance subsampling. The random forest and bagging GLMs also used implicit feature selection, in which random subsets of the feature space were used to train classifiers which were then combined to produce an aggregate prediction.

We analyzed overall model performance in predicting our dichotomized HRQOL measure by conducting receiver operating characteristic (ROC) curve analysis. We evaluated resulting models using a held-out test set of 20% of the dataset. We generated accuracy scores, confusion matrices, and ROC curves for each test. Our main analysis was performed using data from subjects recruited from Cint or Cedars-Sinai. A follow-up analysis was also performed combining this data with data obtained from the University of Pennsylvania.

## Results

### Participant Characteristics

Table 2 presents demographic information of the participants recruited from Cedars-Sinai, Cint, and the University of Pennsylvania. Because some users signed up multiple times through multiple accounts, we removed duplicate entries from the study. In addition, we removed participants who had protected (i.e., non-public) Twitter accounts that could not be analyzed, or who had zero tweets in the analysis period. In the end, recruitment efforts yielded 1,831 users who completed the survey at enrollment.. We could retrieve at least one tweet from 835 of the original 1,831 subjects. The remaining 996 subjects either did not tweet during the 60-day period or had a protected or deleted twitter account when tweet collection was being performed. Of the 835 subjects with tweets, 581 of them completed both surveys, and the remaining 254 only completed the first survey. The latter subjects were discarded from the analysis. In the follow-up analysis, data obtained from the University of Pennsylvania for 43 patients (with accessible Tweets) was added to the data from Cedars-Sinai and Cint.

Over each 30-day period, participants posted an average of 99 messages (range 0 to 4880). The rate at which patients posted did not significantly differ between the pre-enrollment period and post-enrollment period (p=0.19). The initial tweet corpus had neutral sentiment overall, as measured by the difference of the average positive and negative SentiStrength score across all Tweets in this analysis (M(Pos) = 1.58, SD(Pos) = 0.81, M(Neg) = 1.43, SD(Neg) = 0.84).

### Predictive Model Accuracy

**Figure 1 -:**
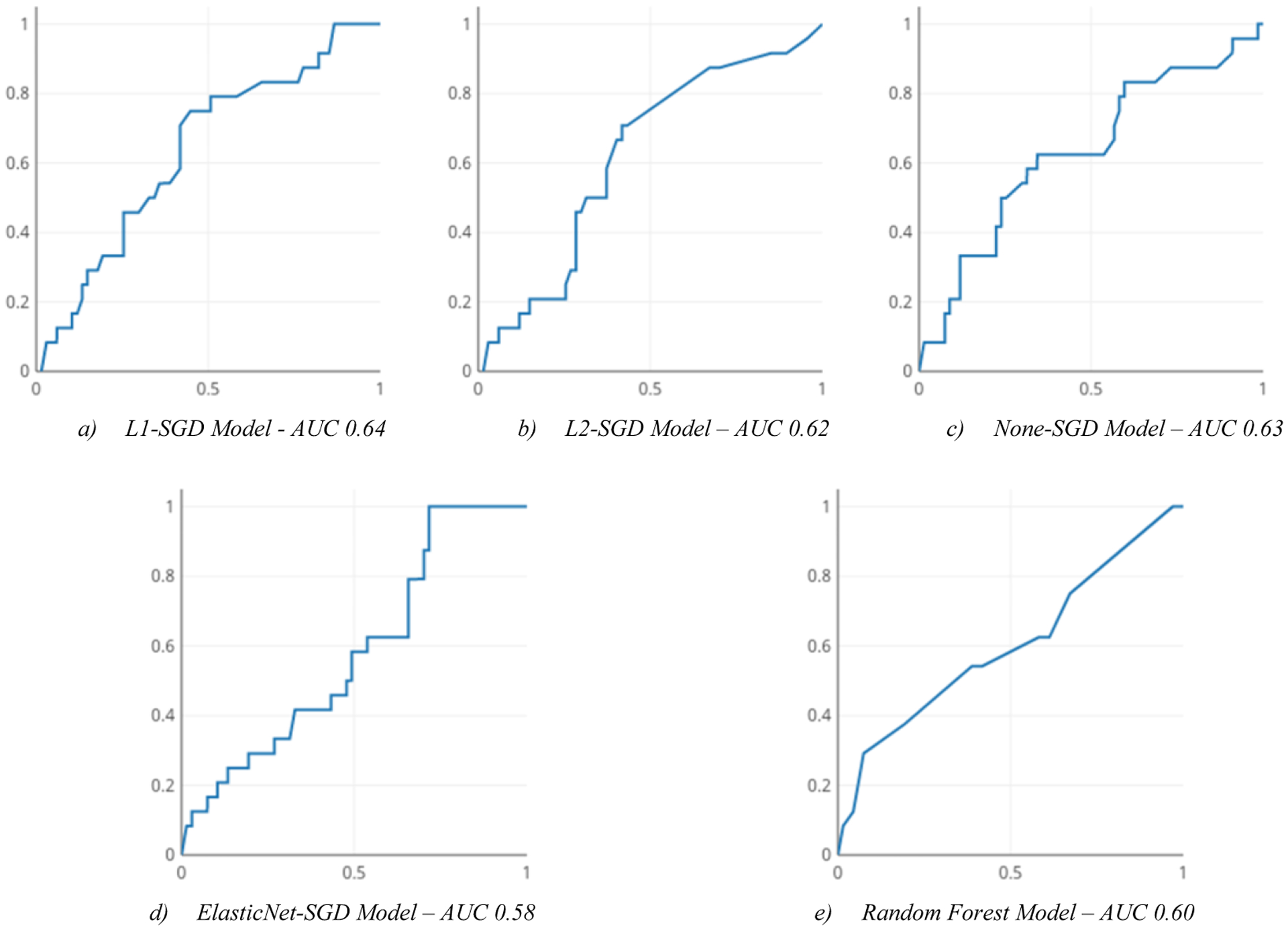
Best ROC Curves for Five Classes of Prediction Models. Models evaluated included bagging generalized linear models using a) L1 regularization, b) L2 regularization, c) no regularization, or d) ElasticNet combination regularization, as well as e) a random forest model. The best performance was obtained from the bagging GLM model using L1 regularization, with an AUC of 0.64. This model used 100 estimators with a maximum sample and feature proportion of 0.75 with bootstrapping, and was trained with 500 iterations of SGD. As can be seen from the ROC curves, the three standard logistic regression models outperformed the random forest model and the combination ElasticNet model.

To develop our model, we employed a grid search to test various combinations of model designs and subsets of our data. The tested model designs included generalized linear models with varying forms of regularization, as well as a random forest model. We also tested subsetting our data to include or exclude each of the three data sources (Cedars-Sinai, Cint, and UPenn). We also tested different methods of handling subjects who completed both surveys, including using only the first collection wave and survey, and using both collection waves and surveys. The resulting models achieved AUCs ranging from 0.58 to 0.64 for predicting the ground truth binary HRQOL status (Figure 1). The highest AUC of 0.64 was achieved using a bagging GLM model with L1 regularization, using all three datasets and all available collection waves. The model used 100 estimators with a maximum sample and feature proportion of 0.75 with bootstrapping, and was trained with 500 iterations of SGD. Further optimization of logistic regression parameters, model design and feature selection, or tweet subsets was unable to achieve a greater AUC than 0.64.

## Discussion

The goal of this study was to develop a PRO instrument that estimates HRQOL from a user’s social media posts on Twitter. Further, we studied the feasibility and validity of measuring HRQOL without prompting users to talk specifically about health-related issues. After using a wide range of text processing methods, the best achievable AUC in predicting ground-state HRQOL was 0.64. Although this performance may be considered sub-optimal, it is better than chance and suggests there is at least a correlation between the language used in Tweets and actual HRQOL; a notable finding considering that the data collected was not posted with the intent to provide insight into the HRQOL status of the participants. However, the results also indicate that free-range social media data only offer a window into HRQOL, but do not afford direct access to current health status. Despite the inherent limitations in this pragmatic, naturalistic study, our classification algorithm exhibited moderate performance in estimating true HRQOL status (i.e. high vs. low using CDC-4 as the gold standard) with an AUC of 0.64 by evaluating the language used in 140 character Tweets, the allowable size of microposts during the time this study was conducted. Of note, Twitter currently allows 240 characters, so it is possible that longer posts could include more health-related data and demonstrate a stronger relationship with ground-truth HRQOL.

We hypothesize that the sub-optimal performance of our classifier is due to a poor signal to noise ratio in the dataset. Most people do not routinely post about their health status, including patients undergoing active care (e.g. the ED patients in this sample). In addition, the subjects from the Cint cohort may be more likely than the baseline Twitter user to tweet about marketing-related subjects rather than personal subjects, because they are paid Twitter marketers for Cint. Considering that subjects in this study were obtained from a variety of sources, received no instructions about what to post, included a combination of patient and general population samples, and were limited to a maximum of 140 characters at a time per tweet, the ability to predict HRQOL above chance suggests that this approach – albeit imperfect – still has merit. Despite the inherent limitations in this preliminary study, our classification algorithm exhibited moderate performance in estimating true HRQOL status (i.e. high vs. low using CDC-4 as the gold standard) with an AUC of 0.64 by evaluating the language used in 140-character Tweets.

Other investigators have encountered sub-optimal performance using social media data to estimate health status. For example, Nascimento and colleagues [9] note that their automated processes for identifying “migraine” versus descriptions of an actual patient’s migraine experiences were difficult to separate. Indeed, in much of our own dataset, even topics generated using NLP that appeared to be health-related were often about commerce (i.e., other users should consider buying product X, rather than the user attesting to how product X treated their condition). Future research could attempt to more specifically recruit “high-volume” non-commercial Twitter users in order to obtain richer features that maybe more predictive, though this may compromise the generalizability of the method. In addition, future efforts could aim to use a larger patient-specific cohort via targeted recruitment, rather than a general population sample. Nevertheless, the challenge of gaining health insights from unstructured “free-range” social media posts is formidable; additional studies are needed to assess the potential for this data to achieve sufficient accuracy for real world clinical and interventional applications.

## Conclusions

In conclusion, we found that analysis of free-range social media posts can predict HRQOL better than chance, but that this technique remains an imperfect method for assessing current health status despite testing a wide range of text processing methods. The best performing model (AUC = 0.64) was a bagging GLM with L1 regularization; this may be helpful when selecting among semantic processing techniques. We hope this study may serve as a template for future research in extracting health data from unstructured social media posts, and believe future studies could improve upon our work by refining recruitment efforts to avoid commercial accounts, expanding the cohort size, and making use of a broader spectrum of social media data from additional platforms.

## Figures and Tables

**Table 1 –:** Selected LDA-generated Topics and Highly Related Tweets

Topic Words (First Three)	Example Tweet
data, health, gt	@[redacted] Yes, we needs a digital platform between patients & medical professional to work 4 better outcome with medication / treatment
t, patient, patients	T4 Patients who are actively engaged should get priority but if a patient wants they should go. 5% of attendees! ideally. #hchlitss
cell, sickle, does	@[redacted] Sickle Cell Disease: underfunded, underresourced, underappreciated
lupus, lupuschat, w	@[redacted] I am in DC to advocate for #Lupus Research. From Culver City to Capitol Hill, here to share my journey of 33 years.

**Table 2 –:** Demographic Characteristics of Twitter Users in Study

Demographics	Value (Cint/CSHS)	Value (UPenn)
Number of Subjects	835	43
Age (Mean ± SD)	40.0 ± 12.6	26.9 ± 7.4
Gender (%)		
*Male*	31.6%	27.9%
*Female*	68.1%	72.1%
*Transgender/Other*	0.3%	0%
Race (%)		
*American Indian / Alaskan Native*	3.0%	0%
*Asian*	5.6%	0%
*Native Hawaiian /Pacific*	0.4%	0%
*Islander*		
*Black or African-American*	11.9%	62.8%
*White*	76.6%	34.9%
*Other / Unknown / Declined to answer*	2.5%	0%

## References

[1] Tweet MS, Gulati R, Aase LA, and Hayes SN, Spontaneous Coronary Artery Dissection: A Disease-Specific, Social Networking Community-Initiated Study, Mayo Clin. Proc 86 (2011) 845–850. doi:10.4065/mcp.2011.0312. 21878595 PMC3257995

[2] Golder SA, and Macy MW, Diurnal and Seasonal Mood Vary with Work, Sleep, and Daylength Across Diverse Cultures, Science (80-.) 333 (2011). doi:10.1126/science.1202775. 21960633

[3] Thaker SI, Nowacki AS, Mehta NB, and Edwards AR, How U.S. Hospitals Use Social Media, Ann. Intern. Med 154 (2011) 707. doi:10.7326/0003-4819-154-10-201105170-00021. 21576547

[4] Weitzman ER, Adida B, Kelemen S, and Mandl KD, Sharing Data for Public Health Research by Members of an International Online Diabetes Social Network, PLoS One. 6 (2011) e19256. doi:10.1371/journal.pone.0019256. 21556358 PMC3083415

[5] Wicks P, Vaughan TE, Massagli MP, and Heywood J, Accelerated clinical discovery using self-reported patient data collected online and a patient-matching algorithm, Nat. Biotechnol 29 (2011) 411–414. doi:10.1038/nbt.1837. 21516084

[6] St Louis C, and Zorlu G, Can Twitter predict disease outbreaks?, BMJ. 344 (2012) e2353. doi:10.1136/bmj.e2353. 22597352

[7] Sinnenberg L, DiSilvestro CL, Mancheno C, Dailey K, Tufts C, Buttenheim AM, Barg F, Ungar L, Schwartz H, Brown D, Asch DA, and Merchant RM, Twitter as a Potential Data Source for Cardiovascular Disease Research, JAMA Cardiol. 1 (2016) 1032. doi:10.1001/jamacardio.2016.3029. 27680322 PMC5177459

[8] De Choudhury M, Counts S, and Horvitz E, Predicting postpartum changes in emotion and behavior via social media, in: Proc. SIGCHI Conf. Hum. Factors Comput. Syst. - CHI ‘13, ACM Press, New York, New York, USA, 2013: p. 3267. doi:10.1145/2470654.2466447.

[9] Nascimento TD, DosSantos MF, Danciu T, DeBoer M, van Holsbeeck H, Lucas SR, Aiello C, Khatib L, Bender MA, Zubieta J-K, DaSilva AF, and DaSilva AF, Real-Time Sharing and Expression of Migraine Headache Suffering on Twitter: A Cross-Sectional Infodemiology Study, J. Med. Internet Res 16 (2014) e96. doi:10.2196/jmir.3265. 24698747 PMC4004155

[10] Eichstaedt JC, Smith RJ, Merchant RM, Ungar LH, Crutchley P, Preoţiuc-Pietro D, Asch DA, and Schwartz HA, Facebook language predicts depression in medical records., Proc. Natl. Acad. Sci. U. S. A 115 (2018) 11203–11208. doi:10.1073/pnas.1802331115. 30322910 PMC6217418

[11] Eichstaedt JC, Schwartz HA, Kern ML, Park G, Labarthe DR, Merchant RM, Jha S, Agrawal M, Dziurzynski LA, Sap M, Weeg C, Larson EE, Ungar LH, and Seligman MEP, Psychological Language on Twitter Predicts County-Level Heart Disease Mortality, Psychol. Sci 26 (2015) 159–169. doi:10.1177/0956797614557867. 25605707 PMC4433545

[12] Reece AG, Reagan AJ, Lix KLM, Dodds PS, Danforth CM, and Langer EJ, Forecasting the onset and course of mental illness with Twitter data, Sci. Rep 7 (2017) 13006. doi:10.1038/s41598-017-12961-9. 29021528 PMC5636873

[13] Schwartz HA, Eichstaedt JC, Kern ML, Dziurzynski L, Lucas RE, Agrawal M, Park GJ, Lakshmikanth SK, Jha S, and Seligman MEP, Characterizing geographic variation in well-being using tweets, in: Seventh Int. AAAI Conf. Weblogs Soc. Media, 2013.

[14] Centers for Disease Control and Prevention, Measuring healthy days: Population assessment of health-related quality of life, Atlanta CDC. (2000) 4–6.

[15] Padrez KA, Ungar L, Schwartz HA, Smith RJ, Hill S, Antanavicius T, Brown DM, Crutchley P, Asch DA, and Merchant RM, Linking social media and medical record data: a study of adults presenting to an academic, urban emergency department, BMJ Qual. Saf 25 (2016) 414–423. doi:10.1136/bmjqs-2015-004489. 26464519

[16] Hays RD, Sherbourne CD, and Mazel RM, The RAND 36-Item Health Survey 1.0., Health Econ. 2 (1993) 217–27. http://www.ncbi.nlm.nih.gov/pubmed/8275167 (accessed April 16, 2017). 8275167 10.1002/hec.4730020305

[17] Bird S, Klein E, and Loper E, Natural language processing with Python: analyzing text with the natural language toolkit, 3rd ed., O’Reilly Media, Inc., 2009.

[18] Miller GA, and A. G, WordNet: a lexical database for English, Commun. ACM 38 (1995) 39–41. doi:10.1145/219717.219748.

[19] Paice CD, and D. C, Another stemmer, ACM SIGIR Forum. 24 (1990) 56–61. doi:10.1145/101306.101310.

[20] Thelwall M, Buckley K, and Paltoglou G, Sentiment strength detection for the social web, J. Am. Soc. Inf. Sci. Technol 63 (2012) 163–173. doi:10.1002/asi.21662.

[21] Thelwall M, Buckley K, Paltoglou G, Cai D, and Kappas A, Sentiment in short strength detection informal text, J. Am. Soc. Inf. Sci. Technol 61 (2010) 2544–2558. doi:10.1002/ASI.V61:12.

[22] Thelwall M, Buckley K, and Paltoglou G, Sentiment in Twitter events, J. Am. Soc. Inf. Sci. Technol 62 (2011) 406–418. doi:10.1002/asi.21462.

[23] Blei DM, Ng AY, and Jordan MI, Latent Dirichlet Allocation, J. Mach. Learn. Res 3 (2003) 993–1022.

[24] Savova GK, Masanz JJ, V Ogren P, Zheng J, Sohn S, Kipper-Schuler KC, and Chute CG, Mayo clinical Text Analysis and Knowledge Extraction System (cTAKES): architecture, component evaluation and applications., J. Am. Med. Inform. Assoc 17 (2010) 507–13. doi:10.1136/jamia.2009.001560. 20819853 PMC2995668

